# Imaging overutilisation: Is enough being done globally?

**DOI:** 10.2349/biij.7.1.e6

**Published:** 2011-01-01

**Authors:** B Rehani

**Affiliations:** Department of Radiology, University of Cincinnati, OH

The discovery of X rays by Roentgen in 1895 was one of the greatest discoveries with historical impact on on each and every one of us. The ability to view anatomy and infer function of inner organs and tissues of human body has provided immense potential that have led imaging to therapeutic arena through interventions and follow-up. How much imaging is appropriate is a legitimate question to ask. This stems from recent emphasis on overutilisation of medical imaging [1–2]. Overutilisation of imaging has been defined as any application where imaging is unlikely to improve patient outcome. Being a probabilistic situation the uncertainties of the outcome provide ground for appropriateness. Therefore, a number of prominent organisations such as the American College of Radiology (ACR), Royal College of Radiology (RCR) and World Health Organization (WHO) have provided appropriateness criteria [3–5]. Despite the existence of these criteria, a significant fraction (perhaps 20 to 50 percent in some areas) of radiological examinations may be inappropriate [6]. Large part of the growth in imaging is beneficial and it cannot be considered overutilisation. In a recent summit organised by the American Board of Radiology Foundation (ABRF) in collaboration with the American Board of Radiology and the National Institute of Biomedical Imaging and Bioengineering, it became clear that detailed considerations support the conclusion that overutilisation exists and numerous factors drive it [1]. Some of the factors are: defensive medicine, self-referral, patient wishes, inappropriate financially motivated factors, health system factors, industry, media and lack of awareness. Some publications have discussed these factors [7–8].

What has not been commented on is the difference between developed and developing countries. While overutilisation is becoming a problem for developed countries, lack of access remains an issue in a large part of the third world, despite the fact that there has been an increase in the rate of growth and unnecessary radiation dose to patients, in a number of developing countries, undergoing computed tomography (CT) and interventional procedures [9–10]. Even though access is limited in developing countries, inappropriate utilisation of imaging modalities still exists. While defensive medicine and self-referral are relatively minor or insignificant issues in developing countries, lack of awareness, patient wishes and inappropriate financially motivated factors are dominant causes. In a study from Canada, it was concluded that although patients do not wish to be involved in problem-solving tasks, few wish to hand over decision-making control to their physician [11]. The emphasis in patient’s wishes is increasingly being debated. In developing countries, there are instances when imaging procedures are perceived by patients as being of therapeutic value even if there may be lack of documentation. Thus, some medical professionals deem it beneficial for the patient, on psychological considerations, to subject them to imaging. There is a clear need to establish just how much patient wishes contribute to overutilisation of imaging in developing countries and it would be no surprise if a systematic study found this to play a major role.

Experience and the published literature suggest that in clinical settings, both referring and radiological medical practitioners often have limited awareness of the referral criteria and radiation risks involved [6–7]. The consumerist culture and media-driven aspects are becoming important in developing countries too. Medical tourism is driving patients from developed countries to relatively lesser developed countries that provide medical facilities at a lower cost. Although overuse has not specifically been reported with this group, there is possibility of patient wishes driving increased use of imaging modalities.

The developed countries have utilised stricter controls that require prescription of some examinations only by specifically qualified specialists, supplemented by accreditation and certification mechanisms. Unfortunately, despite their existence, the current situation of overutilisation in developed countries shows that requirements and controls have proven inadequate.

The solution for this problem might better be found in human behavioral approaches. The usage pattern of radiological procedures may well decrease if every doctor while prescribing or performing a radiological examination was to ask himself/herself, would I prescribe this procedure if the patient was my own child? In the absence of a better term, we can call it decision-making based on moral considerations, although some would put this in the category of ethics, which strictly does not cover the moral issue involved. Sadly, such simpler and powerful tools are not propagated as intensively as above-mentioned tools such as appropriateness criteria, accreditation mechanisms, requirements on training, etc.

There is almost certainly merit in returning to traditional medicine ideology, where care is provided to the individual based on subjective clinical judgment. Not withstanding the contribution of objective imaging tools, we undervalue the power of subjective judgments and overvalue objectivity. Applying the same standard objective list of investigations to all patients with a particular kind of symptom thoughtlessly leads to unnecessary imaging. An important question for the referring practitioner to ask would be what radiological imaging is needed and whether the results of the imaging will actually change the management in this particular case? Not that this is not practiced, but reinforcing this approach as much as we depend on accreditation (just to name one) may have higher potential for success in reducing overutilisation.

Perhaps, clinical outcome studies and surveys which compare residents and physicians better trained to utilize the value of their moral and subjective clinical judgments with those not driven by these factors would help in exploring whether this approach aids in decreasing unnecessary radiological examinations.

## References

[1] American Board of Radiology Foundation http://www.abrfoundation.org/summit.html.

[2] International Atomic Energy Agency http://rpop.iaea.org/RPOP/RPoP/Content/PastEvents/over-utilization-medical-imaging.htm.

[3] American College of Radiology ACR Appropriateness Criteria.

[4] Royal College of Radiologists (2007). Making the best use of clinical radiology services, Referral Guidelines.

[5] World Health Organization Effective choices for diagnostic imaging in clinical practice.

[6] Hadley JL, Agola J, Wong P (2006). Potential impact of the American College of Radiology Appropriateness Criteria on CT for trauma. AJR Am J Roentgenol.

[7] Levin DC, Rao VM (2004). Turf Wars in Radiology: The overutilization of imaging resulting from self-referral. J Am Coll Radiol.

[8] Emanuel EJ, Fuchs VR (2008). The perfect storm of overutilization. JAMA.

[9] Tsapaki V, Ahmed NA, AlSuwaidi JS, Beganovic A, Benider A, BenOmrane L, Borisova R, Economides S, El-Nachef L, Faj D, Hovhannesyan A, Kharita MH, Khelassi-Toutaoui N, Manatrakul N, Mirsaidov I, Shaaban M, Ursulean I, Wambani JS, Zaman A, Ziliukas J, Zontar D, Rehani MM (2009). Radiation exposure to patients during interventional procedures in 20 countries: initial IAEA project results. AJR Am J Roentgenol.

[10] Muhogora WE, Ahmed NA, Beganovic A, Benider A, Ciraj-Bjelac O, Gershan V, Gershkevitsh E, Grupetta E, Kharita MH, Manatrakul N, Milakovic M, Ohno K, Ben Omrane L, Ptacek J, Schandorf C, Shabaan MS, Stoyanov D, Toutaoui N, Wambani JS, Rehani MM (2009). Patient doses in CT examinations in 18 countries: initial results from International Atomic Energy Agency projects. Radiat Prot Dosimetry.

[11] Deber RB, Kraetschmer N, Irvine J (1996). What role do patients wish to play in treatment decision making?. Arch Intern Med.

